# Promotive and preventive interventions for mental health and well-being in adult populations: a systematic umbrella review

**DOI:** 10.3389/fpubh.2023.1201552

**Published:** 2023-08-31

**Authors:** Maija Saijonkari, Elsa Paronen, Timo Lakka, Tommi Tolmunen, Ismo Linnosmaa, Johanna Lammintakanen, Jenni Isotalo, Hanna Rekola, Tomi Mäki-Opas

**Affiliations:** ^1^Department of Health and Social Management, University of Eastern Finland, Kuopio, Finland; ^2^Institute of Biomedicine, School of Medicine, University of Eastern Finland, Kuopio, Finland; ^3^Department of Clinical Physiology and Nuclear Medicine, Kuopio University Hospital, Kuopio, Finland; ^4^Foundation for Research in Health Exercise and Nutrition, Kuopio Research Institute of Exercise Medicine, Kuopio, Finland; ^5^Kuopio University Hospital, Kuopio, Finland; ^6^Department of Public Health and Welfare, National Institute for Health and Welfare, Helsinki, Finland; ^7^Department of Social Sciences, University of Eastern Finland, Kuopio, Finland; ^8^Wellbeing Services Research Center, North Savo Wellbeing Services County, Kuopio, Finland

**Keywords:** mental health, mental wellbeing, alcohol, promotion, prevention, cognitive behavioral therapy, effectiveness, healthy lifestyle

## Abstract

**Introduction:**

Mental health disorders are increasing worldwide, leading to significant personal, economic, and social consequences. Mental health promotion and prevention have been the subject of many systematic reviews. Thus, decision makers likely face the problem of going through literature to find and utilize the best available evidence. Therefore, this systematic umbrella review aims to evaluate the effectiveness of interventions for promoting mental health and mental well-being, as well as for the primary prevention of mental health disorders.

**Methods:**

Literature searches were performed in APA PsycInfo, Medline, and Proquest Social Science databases from January 2000 to December 2021. The search results were screened for eligibility using pre-defined criteria. The methodological quality of the included reviews was evaluated using the AMSTAR 2 tool. The key findings of the included reviews were narratively synthesized and reported with an emphasis on reviews achieving higher methodological quality.

**Results:**

Out of the 240 articles found, 16 systematic reviews and four systematic umbrella reviews were included. The methodological quality of included reviews was low or critically low.

**Discussion:**

This review suggests that interventions using cognitive-behavioral therapy and those developing resilience, mindfulness, or healthy lifestyles can be effective in the promotion of mental health and well-being in adult populations. Motivational interviewing may reduce alcohol consumption in young adults. Indicated or selective prevention is likely to be cost-effective compared to universal prevention. Parenting interventions and workplace interventions may be cost-effective in terms of promoting mental health. Due to the low methodological quality of the included reviews and substantial heterogeneity among the reported results, the findings from the reviews we summarized should be interpreted with caution. There is a need for further rigorous, high-quality systematic reviews.

## 1. Introduction

Mental health is defined by the World Health Organization (WHO) as “a state of mental well-being that enables people to cope with the stresses of life, realize their abilities, learn well and work well, and contribute to their community” (1). WHO also states that “mental health is an integral component of health and well-being and is more than the absence of mental disorder” (1). Mental health disorders, which also include substance addictions, are increasing worldwide, and have significant human, economic, and social consequences (2). Although mental problems affect every social class, some disadvantaged groups are particularly vulnerable to them (3). These groups, by definition, are lacking basic resources or conditions necessary for an equal position in society (4).

The COVID-19 crisis has affected negatively the already burdening mental health situation (5). In its recent report (5), the Organization for Economic Co-operation and Development (OECD) stressed the urgent need for integrated, mental health support encompassing the whole of society and identified access to evidence-based mental health promotion programs as one priority. Mental health promotion often refers to interventions aimed at improving positive mental health and well-being, that strengthen and protect mental health and may also prevent mental health disorders (6). Prevention of mental disorders, on the other hand, focuses on the causes and risk factors of mental health disorders. It can be defined as primary, secondary, or tertiary prevention depending on whether the strategy aims at (i) preventing the onset of symptoms or disorder, (ii) reducing the prevalence of the disorder or (iii) reducing the severity, course or duration of the disorder and associated disability, respectively (6). Primary prevention activities can be designed as (i) universal (for the general population), (ii) selective (for high-risk groups), or (iii) indicated (for high-risk individuals displaying symptoms of illness but not meeting full diagnostic criteria) (6–9). The promotion of positive mental health and the primary prevention are overlapping and complementary activities that can be present within the same program (6).

When implementing new approaches for mental health and well-being it is important to prioritize the delivery of effective interventions (10). It is also important to understand for whom the intervention works and under what conditions, to be able to embed new interventions in normal activities and practices in a sustainable way (11, 12).

Mental health promotion and prevention have been the subject of many systematic reviews. Thus, decision makers likely face the problem of going through literature to find and utilize the best available evidence. Some scoping reviews have mapped the body of literature concerning mental health promotion and prevention (13, 14), but comprehensive umbrella reviews for this topic are scarce. Hence, a summary of existing research syntheses related to mental health promotion and prevention interventions for the adult population is needed. The aim of this systematic umbrella review was to evaluate the effectiveness of intervention approaches among adult populations aged 18–64 for:

- Promoting mental health and mental well-being, as well as,
- Primary prevention of mental health disorders, including substance abuse problems.

In addition, we aimed to identify the cost-effectiveness of the interventions as well as factors contributing to the effectiveness of the interventions.

## 2. Methods

This study employed the Joanna Briggs Institute (JBI) umbrella review method (also called review of reviews, overview of reviews), which is an established way of bringing together and summarizing a broad evidence-base utilizing all types of syntheses of research evidence (15). An umbrella review provides a summary of existing research syntheses related to a given topic and does not re-synthesize the results of existing reviews with meta-analysis or meta-synthesis (15). This review was carried out and reported using the Preferred Reporting Items for Systematic Reviews and Meta-analyses (PRISMA) guideline (16). A completed PRISMA checklist is included in Supplementary Table S1 in the online supplementary materials.

### 2.1. Search strategy

Literature searches (Table 1) were performed in cooperation with social and health sciences information specialist in three databases, APA PsycInfo, Medline, and ProQuest Social Sciences. The keywords used in the searches were: “mental health,” “wellbeing” “well-being,” “psych* well-being,” “mental illness,” “substance abuse,” “alcohol,” “tobacco,” “drug*,” “promot*,” “prevent*,” “intervention,” “program.” Search limiters that were used (when available) included systematic reviews published between January 2000 to December 2021, and human studies.

**Table 1:** Search strategy.

Database	Search strategy	Search result
APA PsycInfo (EBSCOhost)	S1: TI (“mental health” OR “psych* well-being”) AND TI promot* AND (program OR intervention) Limiters - Publication Year: 1950–2021, Methodology: meta-analysis or systematic review or literature review.	
	S2: TI (“mental health” OR “psych* well-being”) AND TI promot* AND (program OR intervention) AND TI review* Limiters - Publication Year: 1950–2021.	
	S1 OR S2 Limiters – Publication Year: 2000–2021	66
Social Sciences (ProQuest)	S1: ti ("mental health" OR "psych* well-being") AND ti (promot*) AND noft (program OR intervention). Limiters: (“Literature Review” OR “Review” OR “Evidence Based Healthcare”) AND PEER(yes)	
	S2: ti("mental health" OR "psych* well-being") AND ti(promot*) AND noft(program OR intervention). Limiters: (“Literature Review” OR “Review” OR “Evidence Based Healthcare”) AND PEER(yes)	
	S1 OR S2 Limiters applied: 2000–2021	29
Pubmed (Medline)	(“mental health”[Title] OR “psychological well-being”[Title]) AND promot*[Title] AND (program OR intervention) Filters: Meta-Analysis, Review, Systematic Review, Humans, from 2000–2021	103
	(“mental illness”[Title] OR “substance abuse”[Title] OR alcohol[Title] OR tobacco[Title] OR drug*[Title]) AND (prevent*[Title]) AND (program[Title] OR intervention[Title]) Filters: Meta-Analysis, Review, Systematic Review, Humans, from 2000–2021	25
		Total 230

In addition, articles identified through relevant reviews were also considered, and the reference lists of the selected articles were checked to identify publications that might not have been found in the search.

### 2.2. Study selection and quality appraisal

The title and abstract of articles as well as the full text of potentially relevant articles were screened against pre-defined eligibility criteria (Table 2) by two independent reviewers (MS, JI). Consensus on article inclusion was reached via discussion.

**Table 2:** Inclusion and exclusion criteria.

	Inclusion criteria	Exclusion criteria
Population	Non-clinical population	A specific group of patients (e.g., mental health promotion among cancer patients).
	Aged 18–64 years (majority of study participants).	–
Intervention	Promotion of mental health or mental wellbeing or primary prevention of mental health disorder or substance abuse.	Treatment of mental health disorder or substance abuse.
Comparison	Systematic review included mainly studies with controls; any alternative approach to support mental health, or no intervention.	–
Outcome	Any measurable indicator of mental health, mental wellbeing or substance use/substance use habits.	-
	Success factor or cost data of the intervention.	
Setting	Community (not health care units)	Health services unit
	Western countries (Europe, United States, Canada, Australia, New Zealand)	Non-Western countries
Follow-up	At least one month	Less than one month follow-up
Publication time	2000–2021	–

Following the criteria of the Database of Abstracts of Reviews of Effects (DARE), used in previous umbrella reviews (17, 18), a review was deemed systematic if it fulfilled four of the following five criteria with Criteria 1–3 being mandatory: (1) Were inclusion/exclusion criteria reported? (2) Was the search adequate? (3) Were the included studies synthesized? (4) Was the quality of the included studies assessed? (5) Are sufficient details about the individual included studies presented?

An umbrella review was included in this review if it reported the effectiveness of the interventions studied. Otherwise, it was used as a reference source. A review that was already included in one of the umbrella reviews was excluded from this review to avoid giving it too much weight. No separate search was conducted on costs or cost-effectiveness of the interventions and factors contributing to the success of the intervention, but any reported information was collected from studies found in our literature search.

The methodological quality of the included reviews was appraised using the AMSTAR 2 tool (A MeaSurement Tool to Assess systematic Reviews) (19), which has proven its reliability and validity for systematic reviews that include both randomized and observational studies. One reviewer (MS) evaluated the included articles. Another reviewer (JI) independently evaluated five (23%) of the articles for quality control. Discrepancies were resolved by consensus. Inter-rater reliability was calculated with percentage of agreement between reviewers.

### 2.3. Data extraction and synthesis

The following data were extracted from the included reviews: title, study type, amount, and type of included studies, population, intervention, main findings relevant to this review (data on mental health, mental well-being, substance use/substance use habits of the study participants, or success factors or cost data of the interventions), time of searches, and funding sources. Data was extracted and tabulated by one reviewer (MS) and checked in full by another (JI).

Key findings of included reviews were narratively synthetized by population type with evidence from higher methodological quality reviews reported in greater detail (15).

## 3. Results

**Figure 1:**
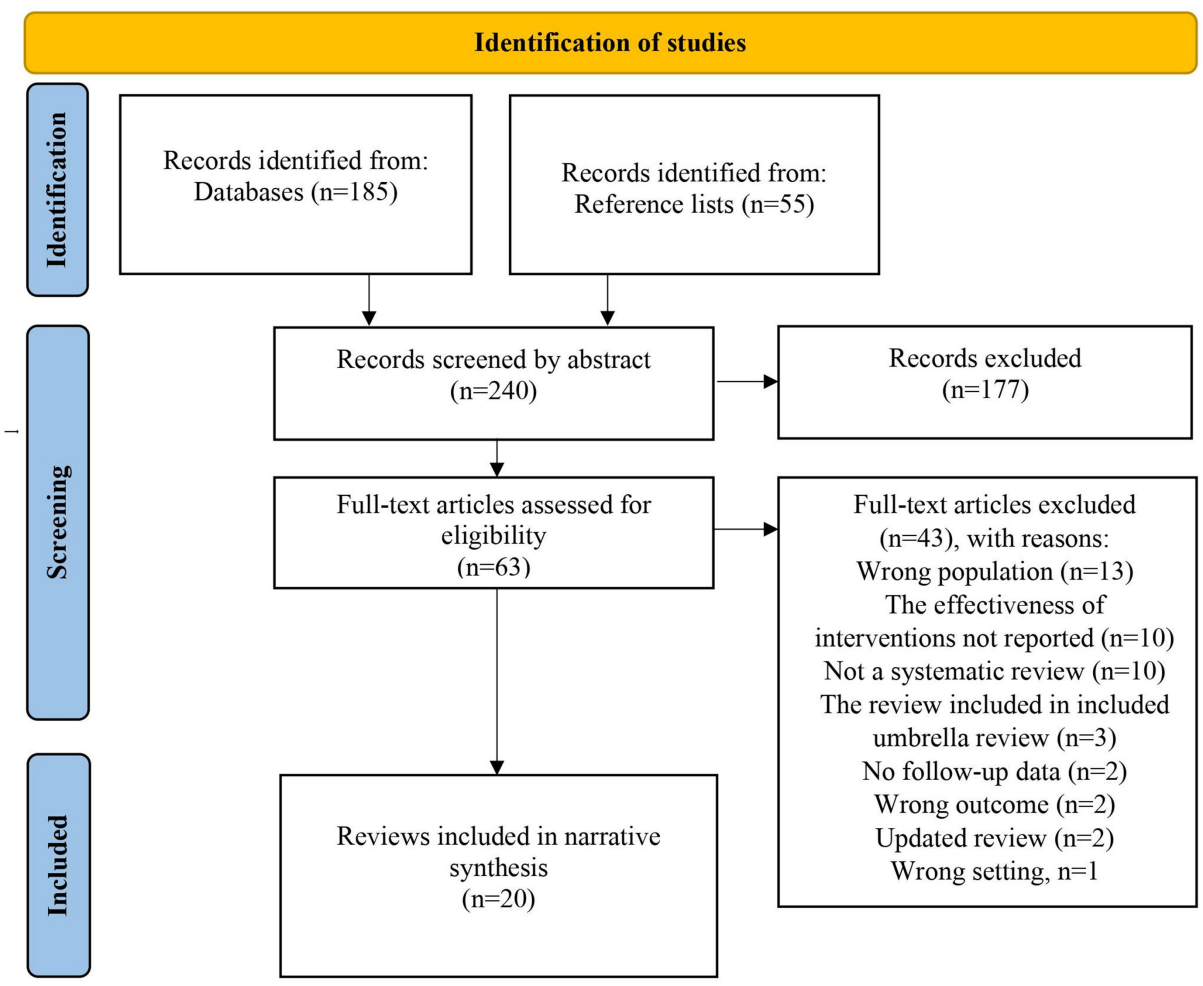
Flow chart of the process of identifying and selecting studies (16).

Literature searches yielded 240 papers of which 63 were read in full text. Of these, 43 articles were excluded (Supplementary Table S2 in the online supplementary materials) for reasons outlined in the PRISMA flow chart in Figure 1. Consequently, 20 articles (20–39) were included (see Figure 1 for PRISMA flowchart and details).

### 3.1. Description of included reviews

The included reviews consisted of four systematic umbrella reviews (29–32) and 16 systematic reviews (20–28, 33–39). Seven of the reviews performed a meta-analysis (20, 21, 25, 33, 35–37). Of these 20 reviews, 18 addressed the effectiveness of mental health promotive and preventive interventions (20–23, 25–38), one the cost-effectiveness of such interventions (24), and one the effectiveness and cost-effectiveness of such interventions (39).

### 3.2. Quality of included reviews

Critical appraisal using the AMSTAR 2 criteria (Supplementary Table S3) revealed one weakness out of the seven possible critical domains for nine reviews (20, 21, 24, 28, 33, 34, 36, 37, 39) meaning their methodological quality was low. Five reviews had two (22, 23, 27, 29, 30), four had three (25, 26, 31, 38) and two had four (32, 35) weaknesses in critical domains, pointing toward critically low methodological quality. The agreement between reviewers concerning five articles assessed in duplicate was 95%.

Across reviews, there were no or only slight weaknesses regarding the comprehensiveness of the literature search strategy (domain 4) (Supplementary Table S3). Regarding the appropriateness of risk of bias assessment (domain 9), 16 reviews had no weaknesses (20–30, 33, 34, 36, 37, 39). On the contrary, only two reviews had no weaknesses regarding the reporting of excluded studies (domain 7) (20, 28) and eight reviews regarding *a priori* design (domain 2) (20, 21, 24, 28, 33, 34, 36, 37, 39). Critical domains 11 and 15 concerning statistical methods and publication bias, respectively, were not relevant in 13 reviews that did not perform a meta-analysis (22–24, 26–32, 34, 38, 39).

### 3.3. The effectiveness of interventions

Of the 19 reviews addressing the effectiveness of the interventions, five covered young adults (aged 18–25 years) (25, 31, 34–36), one parents and families (20), five employees at work-places (21, 28–30, 32), two disadvantaged groups (22, 38), and six the general adult population (23, 26, 27, 33, 37, 39). A meta-analysis was performed in seven of these reviews (20, 21, 25, 33, 35–37).

#### 3.3.1. Interventions for young adults

We identified four systematic reviews (25, 34–36) and one umbrella review (31) on the impact of mental health promotion and prevention interventions for young adults aged 18–25 years (Table 3).

**Table 3:** Characteristics and main findings of included reviews concerning interventions for young adults.

Publication; Study type; Amount and type of included studies (Search’s time span)	Target group	Intervention(s) reviewed	Main findings	Overall methodological quality rating; Weaknesses in critical domains	Funding sources of the review
Dawson, 2020 (36) systematic review and meta-analysis *k* = 40; RCTs (Until March 2017)	University students	Mindfulness-based interventions (MBI)	SMD −0.32; 95% CI −0.50 to −0.13; *p* = 0.0007 for distress and SMD 0.53; 0.33 to 0.73; *p* < 0.00001 for MBI compared to passive controls at three months follow-up. Compared to active control groups (e.g., relaxation or self-awareness strategies), no follow-up data available. The low methodological quality of most of the included trials precludes making firm recommendations for practice, and the variability of the effects means that some students in some contexts may not benefit from MBIs.	12; 1	NR
Lo, 2018 (25) systematic review and meta-analysis *k* = 24; RCTs (Until April 2016)	Health profession students	Group interventions designed to enhance/maintain mental health	CBT interventions reduced anxiety (SMD −0.26; 95% CI −0.5 to −0.02), depression (SMD −0.29; 95% CI −0.52 to −0.05) and stress (SMD 0.37; 95% CI −0.61 to −0.13). Mindfulness strategies reduced stress (SMD −0.60; 95% CI 0.97 to −0.22) but not anxiety (95% CI −0.21 to 0.18), depression (95% CI −0.36 to 0.03) or burnout (95% CI −0.36 to 0.10). Relaxation strategies reduced anxiety (SMD -0.80; 95% CI −1.03 to −0.58), depression (SMD −0.49; 95% CI −0.88 to −0.11) and stress (SMD −0.34; 95% CI −0.67 to −0.01). Method quality was generally poor.	10; 3	NR
Clarke, 2015 (34) systematic review *k* = 28; RCTs and quasi-experimental studies (Jan. 2000–June 2013)	Youth (majority over 18 years of age)	Online Youth Mental Health Promotion and Prevention Interventions	The evidence regarding mental health promotion gaming interventions is weak, as a result of the absence of a control group and high dropout rates in the two studies reviewed.	9; 1	Inspire Ireland Foundation and Young and Well Cooperative Research Centre, Australia.
			Online prevention interventions: promising evidence regarding computerized CBT interventions and their impact on emerging adults’ anxiety and depression symptoms.		
Conley, 2015 (35) systematic review and meta-analysis *k* = 90; RCTs, quasi-experimental (Until Dec. 2012)	Higher Education Students	Universal mental health promotion interventions	Interventions with supervised skills practice: a significant positive effect at follow-up (median 12 weeks) (ES = 0.28, CI = 0.16 to 0.40; *k* = 16), whereas psychoeducational interventions did not (ES = 0.08, CI = −0.04 to 0.21; *k* = 10). The mean ES for the four studies of skills-training interventions without supervised practice was not significant at follow-up (ES = 0.13, CI = −0.14 to 0.39).	4; 4	Loyola University Chicago
Sandler, 2014 (31) review of meta-analytic reviews *k* = 4 relevant reviews (of total of 48 reviews) (2000–2013)	College students up to age of 26	Prevention and promotion programs to prevent alcohol use	Motivational interviewing, blood alcohol content education, normative comparison, and feedback on consumption: small, significant effects on alcohol use and alcohol-related problems at short-term follow-up. Significant effect on frequency of drinking days and alcohol-related problems up to four years after intervention. Heterogeneous effects on alcohol-related problems at short-term follow-up, other effects homogeneous.	5; 3	NR
			Face-to-face interventions: small, significant effect on alcohol use at three- and six-month follow-ups. Motivational interviewing and personalized feedback for heavy drinkers: large significant effects on alcohol consumption and alcohol problems one year after participation. The effects for both outcomes were heterogeneous.		
			Meta-analysis of 14 trials of programs that challenged alcohol expectancies: small, significant effects at post-test, but the effects were non-significant at follow-ups greater than a month.		

CBT, cognitive behavioral therapy; CI, confidential index; *k*, number of studies; NR, not reported; RCT, randomized controlled trial; SMD, standard mean difference.

Dawson et al. (36) included 40 randomized controlled trials (RCTs) of mindfulness-based interventions for university students and found a small but statistically significant effect on distress and a moderate effect on mindfulness compared to no intervention over 3 months. Compared to active control conditions, which typically utilize alternative interventions, no results about follow-up data were available. The authors stressed the low methodological quality of most of the included studies and the variability of the effects. On the other hand, the review by Lo et al. (25) including 24 RCTs found that mindfulness strategies reduced stress but not anxiety, depression, or burnout among health professional students. In addition, cognitive-behavioral interventions showed a significant positive effect on anxiety, depression, and stress, and relaxation-strategy interventions on anxiety, depression, and stress. Again, the quality of included trials was generally poor.

Clarke et al. (34), summarizing 28 RCTs and observational studies, found promising evidence for computerized cognitive behavioral therapy interventions for the prevention of anxiety and depression in emerging adults. The evidence regarding mental health promotion gaming interventions is weak.

Conley et al. (35) included 90 RCTs and quasi-experimental studies on mental health promotion among higher education students. Interventions with supervised skills practice had a significant positive effect on mental health, whereas psychoeducational interventions and skills-training interventions without supervised practice had a nonsignificant effect.

Sandler et al. (31) summarized four meta-analytic reviews of prevention and promotion programs to prevent alcohol use among college students, and found that motivational interviewing, blood alcohol content education, normative comparison, and feedback on consumption have small, statistically significant, but partly heterogeneous effects on alcohol use and alcohol-related problems at short-term follow-up. The effects diminished over time, but the effect on frequency of drinking days and alcohol-related problems remained significant and were homogenous up to 4 years after the intervention. Motivational interviewing and personalized feedback for heavy drinkers had large significant effects of reduced alcohol consumption and alcohol problems 1 year after participation, but the effects on both outcomes were heterogeneous. Programs that challenged alcohol expectancies had no significant effects at follow-ups greater than a month.

Overall, statistically significant beneficial effects were found for mindfulness-based interventions on mindfulness, distress, and stress and for computerized or group-based cognitive behavior techniques, as well as for relaxation strategies on anxiety, depression, and stress among young adults. However, the findings are limited due to the low methodological quality and insufficient number of included primary studies, and the variability of the results. Skills-based mental health promotion interventions with supervision had a significant effect on overall mental health among young adults. Motivational interviewing and personalized feedback were effective in reducing alcohol consumption and alcohol problems.

#### 3.3.2. Interventions for parents and families

We identified one systematic review of mental health promoting interventions for parents and families (20) (Table 4).

**Table 4:** Characteristics and main findings of included reviews concerning interventions for parents and families.

Publication; Study type; Amount and type of included studies (Search’s time span)	Target group	Intervention(s) reviewed	Main findings	Overall methodological quality rating; Weaknesses in critical domains	Funding sources of the review
Barlow, 2014 (20) systematic review and meta-analysis *k* = 48; RCTs (Until 2011)	Parents	Group-based behavioral, cognitive-behavioral or multi-modal parenting program	Statistically significant short-term (2–6 months) improvements in parental depression (standardized mean difference (SMD) -0.17, 95% confidence interval (CI) −0.28 to −0.07), anxiety (SMD −0.22, 95% CI −0.43 to −0.01), stress (SMD −0.29, 95% CI −0.42 to −0.15), anger (SMD −0.60, 95% CI −1.00 to −0.20), guilt (SMD −0.79, 95% CI −1.18 to −0.41), confidence (SMD −0.34, 95% CI −0.51 to −0.17) and satisfaction with the partner relationship (SMD -0.28, 95% CI -0.47 to −0.09). However, only stress and confidence continued to be statistically significant at six-month follow-up, and none were significant at one year. There was no evidence of any effect on self-esteem (SMD −0.01, 95% CI −0.45 to 0.42).	14; 1	UK Cochrane Centre. The University of Warwick, UK. The Institute of Mental Health, Nottingham, UK. NHS Cochrane Programme Grant Scheme, UK.

CI, confidential index; *k*, number of studies; RCT, randomized controlled trial; SMD, standard mean difference; UK, United Kingdom.

Barlow et al. (20) included 48 RCTs and concluded that group-based behavioral, cognitive-behavioral, or multi-modal parenting programs improve parental depression, anxiety, stress, anger, guilt, confidence, and satisfaction with the partner relationship statistically significantly at 2–6 months follow-up. Programs were effective at 6 month follow-up in relieving stress and improving confidence but effects on all outcomes disappeared at 1 year follow-up. No effects on self-esteem were found.

Overall, group-based behavioral, cognitive-behavioral, or multi-modal parenting programs were found to improve parental mental health in the short term.

#### 3.3.3. Workplace interventions

We identified three systematic umbrella reviews (29, 30, 32) and two systematic reviews (21, 28) of studies exploring effects of mental health promoting interventions at the workplace (Table 5).

**Table 5:** Characteristics and main findings of included reviews concerning interventions at workplace.

Publication; Study type; Amount and type of included studies (Search’s time span)	Target group	Intervention(s) reviewed	Main findings	Overall methodological quality rating; Weaknesses in critical domains	Funding sources of the review
Otto, 2021 (28) systematic review *k* = 3 relevant RCTs (of total of 6) (Until Nov. 2020)	Nursing staff in older adult care	1. Physical activity interventions	First positive effects can be demonstrated concerning CBT interventions and multicomponent interventions.	10; 1	No external funding
		2. CBT interventions	There is no strong evidence for any type of intervention affecting physical and mental health. The heterogeneity of the studies regarding all aspects of the interventions and assessed outcome measures makes interpretation more difficult.		
		3. Organizational interventions (resources, working methods, tasks, or the environment)			
Pieper, 2019 (29) review of reviews *k* = 38 relevant reviews (of total of 74) (April 2012 – Oct. 2017)	Male and female employees in different age groups	Workplace interventions (resilience or mindfulness training, CBT, relaxation techniques and organizational-level workplace interventions)	Mindfulness and cognitive-behavioral training as well as peer supervision appeared to help reduce stress. Additionally, organizational interventions including reduction of work impact and flexible worktime seemed to lower stress and burn-out-symptoms. Overall, multi-component programs were more effective than single-component interventions. The authors found cognitive-behavioral programs effective at reducing depression, anxiety, and burnout as well as to improve well-being. One moderate-quality review assessed physical training and yoga-interventions and found them effective in the prevention of stress and anxiety.	7; 2	No external funding
Proper, 2019 (30) review of reviews *k* = 6 relevant reviews (of total of 23) (2009–2018)	Working population	Worksite mental health promotion interventions	Based on high-quality reviews, there is strong evidence that workplace psychological interventions, especially those that use e-health and cognitive behavior techniques, yield positive effects on mental health.	8; 2	European Union, in the framework of the Health Program (2014–2020), grant agreement number 761307.
Bartlett, 2019 (21) systematic review and meta-analysis *k* = 23 RCTs (Until 2016)	Employees in the workplace	Mindfulness training delivered in the work context	Workplace-delivered mindfulness training: beneficial effects for anxiety (*g* = 0.62, *p* = 0.001, *I*^2^ = 0), psychological distress (*g* = 0.69, *p* = 0.001, *I*^2^ = 20), sleep (*g* = 0.26, *p* = 0.003, *I*^2^ = 0), mindfulness (*g* = 0.45, *p* = 0.001, *I*^2^ = 54), stress (*g* = 0.56, *p* = 0.001, *I*^2^ = 79) and well-being (*g* = 0.46, *p* = 0.002, *I*^2^ = 66). Beneficial effects for psychological distress, depression, anxiety, and wellbeing also remained stable at three-month follow-up. No conclusions could be drawn from pooled data for burnout due to ambivalence in results, for depression due to publication bias, or for work performance due to insufficient data. The study that reported null results for mindfulness, wellbeing, and engagement following a six-month mindfulness program saw a continuing absence of effect 12 months from baseline.	14; 1	NR
Bhui, 2012 (32) review of reviews *k* = 23 reviews (1990 – July 2011)	Employees in the workplace	Individual, organizational, and mixed interventions on mental health and absenteeism	CBT was the most effective individual targeted intervention for mental health.	6; 4	Department of Health, UK.
			The only organizational intervention to show convincing effects on absenteeism (the main cause of which are anxiety and depression) was physical activity programs.		

CBT, cognitive behavioral therapy; *g*, effect size; *I*^2^, heterogeneity; *k*, number of studies; NR, not reported; RCT, randomized controlled trial; SMD, standard mean difference; UK, United Kingdom.

Bartlett et al. (21) combined the results of 23 RCTs of mindfulness training delivered in the work context and found it beneficial for anxiety, psychological distress, sleep, mindfulness, stress, and well-being compared to active comparators. The authors could not draw conclusions for burnout due to ambivalence in results and for depression due to publication bias.

Otto et al. (28) conducted a systematic review of physical activity, cognitive-behavioral, and organizational interventions among nursing staff in older adult care. Based on three RCTs, the authors found that cognitive-behavioral and multicomponent interventions had positive effects on nurses’ mental health. However, they reported that there are not enough high-quality studies to make firm conclusions about the effectiveness of studied interventions in this target group.

Proper et al. (30) summarized the results of six reviews of mental health promotion interventions at the workplace. They concluded that there was strong evidence based on high quality reviews indicating that the use of cognitive behavior techniques yields positive effects on employees’ mental health. The reports by Pieper et al. (29) including (30) and Bhui et al. (32), including 28 systematic reviews came to the same conclusion. Proper et al. (30) also reported that there was strong evidence regarding e-health interventions. Pieper et al. (29) found physical training and yoga effective in prevention of stress and anxiety. Bhui et al. (32) found that physical activity programs showed convincing positive effects on absenteeism.

Overall, workplace mindfulness training was beneficial in promoting employees’ mental health compared to active comparators. Based on three umbrella reviews, cognitive behavior techniques were effective in mental health promotion.

#### 3.3.4. Interventions for disadvantaged groups

We identified two systematic reviews of studies concerning mental health promotion and prevention interventions for disadvantaged groups (Table 6).

**Table 6:** Characteristics and main findings of included reviews concerning interventions for disadvantaged groups.

Publication; Study type; Amount and type of included studies (Search’s time span)	Target group	Intervention(s) reviewed	Main findings	Overall methodological quality rating; Weaknesses in critical domains	Funding sources of the review
Koopman, 2017 (22) systematic review *k* = 24, RCTs (NR)	Unemployed people	1. Occupational skills training (OST)	5/8 OST studies reported positive effects and 3/8 no effect on mental health	8; 2	NR
		2. Psychological interventions (PSI)	7/9 PSI studies reported positive effects and 2/9 no effect on mental health		
		3. Combined (OST + PSI)	6/6 Combined studies (including two high-quality studies) reported positive effects on mental health.		
Gottlieb, 2011 (38) systematic review *k* = 13, RCTs and cohort studies (1997–2008)	Unemployed people	Pre-employment training (e.g., employment workshops)	Most community-level preventive interventions for unemployed adults suggested long-term effects of pre-employment training on decreasing depressive symptoms and psychological distress among participants, particularly among those depressed at baseline.	6; 3	NIMH (National Institute of Mental Health) grant #R25MH060288–09.
	Homeless people and people living in public housing	Housing interventions	1/4 studies (the largest study): significant improvement in depressive symptoms.		
	Low-income women, mothers, and victims of domestic violence	Anti-poverty programs, parenting programs, shelter programs	3/4 studies: no improvement in depressive symptoms, but an improvement in other markers of psychological distress, including calmness and peacefulness, self-perception of depressive symptoms, paranoia, hostility, and obsessiveness.		
			3/4 interventions demonstrated improvements in depressive symptoms.		

*k*, number of studies; NR, not reported; OST, occupational skills training; PSI, psychological interventions; RCT, randomized controlled trial.

Koopman et al. (22) summarized 24 RCTs on the effectiveness of mental health promotion interventions among unemployed people, while Gottlieb et al. (38) reviewed 11 RCTs and two cohort studies on the impact of contextual interventions on depression. Studies of pre-employment training included in the review of Gottlieb et al. (38) were also included in Koopman et al. (22).

Koopman et al. (22) reported that the evidence was strongest for combined interventions (CI) consisting of psychological interventions that strengthen psychological resilience and vocational skills training aiming at re-employment: all the included CI studies reported positive effects on mental health and two of these studies were of high quality.

Most studies of community-level preventive interventions for unemployed people reviewed by Gottlieb et al. (38) suggested long-term effects of pre-employment training on decreasing depressive symptoms and psychological distress among participants, particularly among those depressed at baseline. Of the four studies focusing on housing interventions for homeless people or people living in public housing, one large study identified a significant improvement in depressive symptoms whereas three studies demonstrated an improvement in other markers of psychological distress. Three of the four other advocacy interventions, including anti-poverty programs and shelter programs, demonstrated improvements in depressive symptoms.

Overall, vocational skills training combined with resilience-building interventions were effective in the promotion of unemployed adults’ mental health. Housing interventions for homeless people, anti-poverty programs and shelter programs, had a beneficial effect on some mental health outcomes.

#### 3.3.5. Interventions for the general adult population

We identified six systematic reviews of interventions promoting mental health of the general adult population (23, 26, 27, 33, 37, 39) (Table 7).

**Table 7:** Characteristics and main findings of included reviews concerning interventions for general adult population.

Publication; Study type; Amount and type of included studies (Search’s time span)	Target group	Intervention(s) reviewed	Main findings	Overall methodological quality rating; Weaknesses in critical domains	Funding sources of the review
Galante, 2021 (37) systematic review and meta-analysis *k* = 136; RCTs (From inception to Aug. 2020)	Any target group	Mindfulness (MBP)	Compared with no intervention, in most but not all scenarios MBPs improved average anxiety (8 trials; SMD = −0.56; 95% CI −0.80 to −0.33; *p*-value <0.001; 95% PI −1.19 to 0.06), depression (14 trials; SMD = −0.53; 95% CI −0.72 to −0.34; *p*-value <0.001; 95% PI −1.14 to 0.07), distress (27 trials; SMD = −0.45; 95% CI −0.58 to −0.31; *p*-value <0.001; 95% PI −1.04 to 0.14), and well–being (9 trials; SMD = 0.33; 95% CI 0.11 to 0.54; *p*-value = 0.003; 95% CI −0.29 to 0.94).	14; 1	National Institute for Health Research (NIHR).
			Compared with nonspecific active control conditions, in most but not all scenarios MBPs improved average depression (6 trials; SMD = −0.46; 95% CI −0.81 to −0.10; *p*-value = 0.012, 95% PI −1.57 to 0.66), with no statistically significant evidence for improving anxiety or distress and no reliable data on well–being. Compared with specific active control conditions, there is no statistically significant evidence of MBPs’ superiority.		
Lampert, 2021 (23) systematic review *k* = 8; observational studies (Until Nov. 2020)	Non-clinical population	Community gardening (gardening activities)	Community gardeners had significantly better health outcomes (life satisfaction, happiness, general health, mental health, and social cohesion) than their neighbors not engaged in gardening activities.	7;2	Instituto de Sau’de Ambiental.
Hunter, 2019 (39) systematic review *k* = 38; RCTs or quasi-experimental studies (NR)	Any target group	Urban green space interventions (greenways, trails and park-based interventions)	Strong evidence for park-based (7/7 studies) and greenway/trail (3/3 studies) interventions employing a dual approach (i.e., a physical change to the urban green space and promotion/marketing programs) on health and wellbeing.	9; 1	WHO Regional Office for Europe. National Institute of Health Research (NIHR).
			Strong evidence for greening of vacant lots (4/4 studies) for health and wellbeing (e.g., reduction in stress).		
Macedo, 2014 (26) systematic review *k* = 13; RCTs and CTs (Until Jan. 2013)	Non-clinical samples of adults	Resilience promotion programs	RCTs: 6/7 statistically significant positive change in resilience, hardiness or resilience surrogates (e.g., coping or self-esteem).	4; 3	CNPq* and FAPERJ*
			CTs: 5/5 statistically significant positive change in resilience or hardiness or regarding only some of the resilience surrogates.		
			Open trial: statistically significant positive change in the levels of stress and depression, but not in well-being and distress		
Mammen, 2013 (27) systematic review *k* = 30; prospective, longitudinal studies (Jan.1976–Dec2012)	Nonclinical sample, 11–100 years	Physical activity (PA) in the prevention of depression.	25/30 studies: a significant, inverse relationship between baseline PA and follow-up depression.	5; 2	The Canadian Institute for Health Research (CIHR).
			5/30 studies: no relationship between PA and subsequent depression		
			4/30 studies: women, and not men, who participated in PA were less likely to report depression at follow-up.		
			Among the studies that found a protective role, the majority were considered high (*k* = 17) or modest (*k* = 6) methodologic quality. Among studies that revealed null effects, three were of modest, one of low and one of high quality.		
Bowler, 2010 (33) systematic review and meta-analysis *k* = 25; RCTs and observational studies (NR)	Any target group	Exposure to natural environment	There was evidence of beneficial effects of activity in a natural environment compared to the synthetic environment in terms of reduced negative emotions such as anger (Hedges *g* = 0.46; 95% CI = 0.23, 0.69), fatigue (*g* = 0.42; 0.07, 0.76) and sadness (*g* = 0.36; 0.08, 0.63) and positive effect on attention (*g* = 0.32; 0.06, 0.58). No statistically significant effects for energy scores (*g* = 0.28; −0.01, 0.57), anxiety (*g* = 0.12; −0.34, 0.58) and tranquility (*g* = 0.39; −0.08, 0.86).	13; 1	Natural England Contract FST20-84-037 to ASP*.
			Beneficial changes (before-after) on feelings of energy ES 0.76 (95% CI 0.30 to 1.22); anxiety 0.52 (0.25, 0.79), significant heterogeneity; anger 0.35 (0.07, 0.64); fatigue 0.76 (0.41, 1.11); and sadness 0.66 (0.16, 1.16)		

CBT, cognitive behavioral therapy; CI, confidential index; *g*, effect size; MBP, mindfulness based program; *k*, number of studies; NR, not reported; PA, physical activity; PI, predictive interval; RCT, randomized controlled trial; SMD, standard mean difference; WHO, World Health Organization. *Abbreviations not explained in the article.

Galante et al. (37) included 136 RCTs on the effectiveness of mindfulness-based programs (MBP) in non-clinical settings. Compared to passive control (no intervention or wait list), MBPs on average had a moderate positive effect on psychological distress, depression, and anxiety, as well as on well-being but to a lesser extent. Compared with taking nonspecific action, MBPs had a moderate positive effect on depressive symptoms and the relationship with the self (e.g., self-esteem, self-compassion). There was no statistically significant evidence for improving anxiety or distress and no reliable data on well-being. When compared with specific active control conditions, no significant evidence for MBPs’ superiority was found. Given the overall high risk of bias in the included trials and the heterogeneity between studies, there was no certainty that the results represent the true effects and that MBPs work in every setting.

Lampert et al. (23), Hunter et al. (39), and Bowler et al. (33) focused on green space interventions. Lampert summarized eight cross-sectional studies and concluded that community gardeners, when compared with their neighbors who were not engaged in gardening activities, had statistically significantly better health outcomes in terms of life satisfaction, happiness, general health, mental health, and social cohesion. Hunter et al. (39), reviewing 38 RCTs or quasi-experimental studies, reported strong evidence to support park-based and greenway/trail interventions employing a dual approach (i.e., a physical change to the urban green space and promotion/marketing programs), as well as interventions related to the greening of vacant lots promoting health and well-being. Based on (30) studies, Bowler et al. (33) found that exposure to the natural environment compared to the synthetic environment reduced negative emotions such as anger, fatigue, and sadness, and had a positive effect on attention. There were no significant effects on energy scores, anxiety, and tranquility.

Mammen and Faulkner (27) conducted a systematic review of 30 prospective studies focusing on physical activity in the prevention of depression. Twenty-five of the studies found a statistically significant, inverse relationship between baseline physical activity and follow-up depression. According to the authors, there is sufficient evidence to conclude that physical activity may prevent depression.

Macedo et al. (26) performed a qualitative synthesis of 13 trials, which reported some degree of improvement in resilience-like variables among populations participating in most resilience-promoting programs. Authors concluded there is evidence pointing towards some degree of effectiveness of resilience promotion programs, despite substantial heterogeneity in study designs and measurements.

Overall, evidence suggests effectiveness of mindfulness-based programs in promoting mental health as well as resilience promotion programs in improving resilience-like variables among the average non-clinical adult population. However, due to the overall high risk of bias and great heterogeneity in the included studies, these conclusions should be interpreted with caution. Green space interventions had beneficial effects on some mental health and well-being outcomes studied. Physical activity prevented the onset of depression.

### 3.4. Cost-effectiveness of the interventions

Two systematic reviews (24, 39) considered cost-effectiveness of mental health promotion and prevention interventions (Table 8).

**Table 8:** Characteristics and main findings of included reviews reporting economic analyses.

Publication; Study type; Amount and type of included studies (Search’s time span)	Target group	Intervention(s) reviewed	Main findings	Overall methodological quality rating; Weaknesses in critical domains	Funding sources of the review
Hunter, 2019 (39) systematic review *k* = 38; RCTs or quasi-experimental studies (NR)	Any target group	Urban green space interventions (park-based interventions, greenways, and trails)	Four studies undertook preliminary economic evaluations and found that urban green space interventions were relatively cost-effective. Cost effectiveness of the three park-based interventions was reported to be $0.14 to $2.40 per Metabolic Equivalent of Task (MET) hours/year (cost effectiveness judged on whether the cost was less than between $0.50 and $1.00 per MET-hour)	9; 1	WHO Regional Office for Europe. National Institute of Health Research (NIHR).
Le, 2021 (24) systematic review *k* = 35 relevant economic studies (of total of 65) (2008–2020)	Adults (18–64)	Mental health promotion and prevention interventions	Targeted (indicated or selective) prevention was likely to be cost-effective compared to universal prevention. Parenting interventions had good evidence in mental health promotion. Strong evidence supported screening plus psychological interventions for mental disorder prevention, while workplace interventions targeting employees in general were cost-effective.	10; 1	National Mental Health Commission, Australia.

*k*, number of studies; MET, metabolic equivalent of task; NR, not reported; RCT, randomized controlled trial; WHO, World Health Organization.

Le et al. (24) summarized evidence of the cost-effectiveness of mental health promotion and prevention interventions from 2008 onwards. The evidence concerning adults aged 18–64 years is based on 35 economic studies, the majority of which achieved fair to high methodological quality. The review found that indicated or selective prevention was likely to be cost-effective compared to universal prevention. Strong evidence supported cost-effectiveness of screening combined with psychological interventions in preventing mental disorders in adults. In addition, workplace interventions targeting employees in general were also considered to be cost-effective. Parenting interventions showed good evidence of cost-effectiveness in mental health promotion. The included return on investment studies, in turn, provided evidence suggesting that preventive interventions for depression and substance abuse in adults produce considerable returns.

Hunter et al. (39) summarized four preliminary economic evaluations of urban green space interventions. Three of the evaluations found interventions to be cost-effective based on the increased physical activity of park users. Authors of the fourth study found increased walking and cycling attributable to investment in trails for walking and cycling and concluded that the investments may have significant benefit–cost ratios. Overall, Hunter et al. (39) concluded, that urban green space interventions aiming to increase physical activity were relatively cost-effective.However, the uncertainties relating to the quality of the included health-economic evaluations likely limits the generalizability of conclusions relating to cost-effectiveness which can be drawn from these two qualitative reviews.

### 3.5. Intervention success factors

Among the key success factors gleaned from this review was the use of supervised practice in universal skills-oriented programs that aimed to promote mental health (35) (Table 9). In the prevention of depression, anxiety, antisocial behavior, and substance abuse, the best results were achieved by programs that used interactive methods to teach the skills needed to bring about the change (31). Methods that engaged participants, such as discussing the materials distributed in the programs and practicing the skills to be taught, also produced better results than simply sharing information (31). Adherence to web-based mental health interventions, which is often poor, could be improved with the provision of face-to-face or online support (34). In studies focusing on alcohol use, largest program effects were achieved for populations with a higher percentage of women; programs delivered face-to-face versus on a computer; and interventions that utilized motivational interviewing, decisional balance exercises, normative feedback, and feedback on expectancies and/or motives for drinking (31).

**Table 9:** Characteristics and main findings of included reviews reporting intervention success factors.

Publication; Study type; Amount and type of included studies (Search’s time span)	Target group	Intervention(s) reviewed	Main findings	Overall methodological quality rating; Weaknesses in critical domains	Funding sources of the review
Clarke, 2015 (34) systematic review *k* = 28; RCTs and quasi-experimental studies (Jan. 2000–June 2013)	Youth (majority over 18 years of age)	Online Youth Mental Health Promotion and Prevention Interventions	Some evidence that participant face-to-face or web-based support is an important feature of online interventions in terms of participant adherence and program outcomes.	9; 1	Inspire Ireland Foundation and Young and Well Cooperative Research Centre, Australia.
Sandler, 2014 (31) review of meta-analytic reviews *k* = 4 relevant reviews (of total of 48 reviews) (2000–2013)	College students up to age of 26	Prevention and promotion programs to prevent alcohol use	Programs that involved more active strategies, such as discussion of the program material and practice of program skills, had larger effects than those that did not include these strategies. Program effects were larger for samples that contained a higher percentage of women; programs delivered in person versus on a computer; and interventions that included motivational interviewing techniques, normative feedback, and feedback on expectancies and/or motives for drinking or a decisional balance exercise. Face-to-face interventions also had greater effects than computer-based interventions in studies that directly compared them.	5; 3	NR

*k*, number of studies; NR, not reported; RCT, randomized controlled trial.

## 4. Discussion

In this systematic umbrella review, evidence was found for the effectiveness of cognitive-behavioral, resilience, mindfulness, and physical activity interventions in promoting mental health and well-being of adult populations. However, the clinical significance of the effects could not be assessed thoroughly, as the umbrella review methodology employed in this review does not allow for a re-synthesis of the results. The effect sizes of the impacts of the interventions could be drawn from eight meta-analytical reviews and are presented in Tables 3–5, 7.

More research literature was found on reducing symptoms of depression and anxiety than on promoting resilience and overall mental well-being, which is in line with a scoping review of Enns et al. (13). However, we found three systematic reviews of resilience interventions (21, 22, 26) published later than the literature search of Enns et al. (13) which points toward a stronger evidence base of resilience interventions in the current literature compared to previous.

The results of this review can be applied to mental health promotion programs targeted at the adult working-age population in Western countries. The preliminary results of this review formed the theoretical framework and development of applied interventions for a mental health promotion program in North Savo, Finland, funded by the European Social Fund. In the future, interventions that prove to be effective during the program will be implemented more widely in the region. As the importance of mental health promotion is likely to increase in the coming years, high quality primary studies and systematic reviews are needed to inform the choice of the most effective interventions. Because of the complexity of the phenomenon, a systemic, multilevel approach is needed to support implementation of the interventions, to monitor their effectiveness, and to involve people and communities in the selection, development, and evaluation of the interventions.

### 4.1. Study strengths and limitations

This systematic umbrella review is relevant to current policymakers and stakeholders, as it evaluated the available evidence of promotive and preventive interventions for mental health and well-being, currently considered a priority in public health. The strengths of this review include the rigorous JBI and PRISMA guidelines, which we followed in carrying out and reporting our work. We included both systematic reviews and umbrella reviews, performed a quality appraisal of included reviews, tabulated the data, and reported the results in as much detail as possible.

The main limitation of this review is the poor methodological quality of the included reviews. The confidence in the results of the included reviews was diminished most often by the lack of a priori design and limited information and justification of the excluded studies. In addition, the methodological quality of the primary studies that were included in the reviews was often poor. Also, when conducting this review, we made some eligibility decisions with undesirably thin data. Thus, some of the included reviews may contain participants in clinical settings or studies with inadequate follow-up time, although we aimed to exclude reviews focusing on participants with a clinical diagnosis as well as studies with less than 1 month of follow-up.

## 5. Conclusion

This review suggests that interventions using cognitive-behavioral therapy and those developing resilience, mindfulness, or healthy lifestyles can be effective in the promotion of mental health and well-being in adult populations aged 18–64. Skills-based mental health interventions with supervision may promote the mental health of young adults and vocational skills training combined with resilience-building interventions may be effective in promoting the mental health of unemployed adults. Motivational interviewing may reduce alcohol consumption in young adults. Indicated or selective prevention are likely to be cost-effective compared to universal prevention. Strong evidence supports the cost-effectiveness of screening combined with psychological interventions in preventing mental disorders in adults. Parenting interventions and workplace interventions may be cost-effective in mental health promotion. Preventive interventions for depression and substance misuse in adults may produce considerable returns on investment. Due to the low quality of the included reviews and the great heterogeneity among the reported results, these conclusions should be interpreted with caution. There is a need for further rigorous, high-quality systematic reviews on promotive and preventive interventions for mental health and well-being. Above all, reviews focusing on the enhancing of mental well-being instead of reducing symptoms of mental problems are needed.

## Data availability statement

The original contributions presented in the study are included in the article/Supplementary material, further inquiries can be directed to the corresponding author.

## Author contributions

MS, EP, TL, TT, IL, JL, and TM-O contributed to conception and design of the study. MS and JI analyzed the data. MS wrote the first draft of the manuscript. TM-O wrote sections of the manuscript. All authors contributed to the article and approved the submitted version.

## Funding

This work was part of the Feel Good North Savo program funded by the European Social Fund (#2014/11114/09020101/2020).

## Conflict of interest

The authors declare that the research was conducted in the absence of any commercial or financial relationships that could be construed as a potential conflict of interest.

## Publisher’s note

All claims expressed in this article are solely those of the authors and do not necessarily represent those of their affiliated organizations, or those of the publisher, the editors and the reviewers. Any product that may be evaluated in this article, or claim that may be made by its manufacturer, is not guaranteed or endorsed by the publisher.
