# Supplementary material for: Promotive and preventive interventions for mental health and well-being in adult populations: a systematic umbrella review
Source: Front Public Health. 2023 Aug 31;11:1201552. doi: 10.3389/fpubh.2023.1201552 (PMC10501832; doi:10.3389/fpubh.2023.1201552)
Supplement: Supplementary file 1 [file Table_1.docx]

## Supplementary Table 1. PRISMA 2020 Checklist^†^

| **Section and Topic** | **Item #** | **Checklist item** | **Location/section of the article where item is reported** |
| --- | --- | --- | --- |
| **TITLE Promotive and preventive interventions for mental health and wellbeing in the adult populations: an umbrella review** | | |  |
| Title | 1 | Identify the report as a systematic review. | Title |
| **ABSTRACT** | | |  |
| Abstract | 2 | See the PRISMA 2020 for Abstracts checklist. | Abstract |
| **INTRODUCTION** | | |  |
| Rationale | 3 | Describe the rationale for the review in the context of existing knowledge. | Section 1 |
| Objectives | 4 | Provide an explicit statement of the objective(s) or question(s) the review addresses. | Section 1 |
| **METHODS** | | |  |
| Eligibility criteria | 5 | Specify the inclusion and exclusion criteria for the review and how studies were grouped for the syntheses. | Section 2.2 |
| Information sources | 6 | Specify all databases, registers, websites, organisations, reference lists and other sources searched or consulted to identify studies. Specify the date when each source was last searched or consulted. | Section 2.2 |
| Search strategy | 7 | Present the full search strategies for all databases, registers and websites, including any filters and limits used. | Section 2.1  and App B |
| Selection process | 8 | Specify the methods used to decide whether a study met the inclusion criteria of the review, including how many reviewers screened each record and each report retrieved, whether they worked independently, and if applicable, details of automation tools used in the process. | Section 2.2 |
| Data collection process | 9 | Specify the methods used to collect data from reports, including how many reviewers collected data from each report, whether they worked independently, any processes for obtaining or confirming data from study investigators, and if applicable, details of automation tools used in the process. | Section 2.3 |
| Data items | 10a | List and define all outcomes for which data were sought. Specify whether all results that were compatible with each outcome domain in each study were sought (e.g. for all measures, time points, analyses), and if not, the methods used to decide which results to collect. | Section 2.3 |
|  | 10b | List and define all other variables for which data were sought (e.g. participant and intervention characteristics, funding sources). Describe any assumptions made about any missing or unclear information. | Section 2.3 |
| Study risk of bias assessment | 11 | Specify the methods used to assess risk of bias in the included studies, including details of the tool(s) used, how many reviewers assessed each study and whether they worked independently, and if applicable, details of automation tools used in the process. | Section 2.2 |
| Effect measures | 12 | Specify for each outcome the effect measure(s) (e.g. risk ratio, mean difference) used in the synthesis or presentation of results. | NA |
| Synthesis methods | 13a | Describe the processes used to decide which studies were eligible for each synthesis (e.g. tabulating the study intervention characteristics and comparing against the planned groups for each synthesis (item #5)). | NA |
|  | 13b | Describe any methods required to prepare the data for presentation or synthesis, such as handling of missing summary statistics, or data conversions. | NA |
|  | 13c | Describe any methods used to tabulate or visually display results of individual studies and syntheses. | NA |
|  | 13d | Describe any methods used to synthesize results and provide a rationale for the choice(s). If meta-analysis was performed, describe the model(s), method(s) to identify the presence and extent of statistical heterogeneity, and software package(s) used. | Section 2.3 |
|  | 13e | Describe any methods used to explore possible causes of heterogeneity among study results (e.g. subgroup analysis, meta-regression). | NA |
|  | 13f | Describe any sensitivity analyses conducted to assess robustness of the synthesized results. | NA |
| Reporting bias assessment | 14 | Describe any methods used to assess risk of bias due to missing results in a synthesis (arising from reporting biases). | NA |
| Certainty assessment | 15 | Describe any methods used to assess certainty (or confidence) in the body of evidence for an outcome. | Section 2.2 |
| **RESULTS** | | |  |
| Study selection | 16a | Describe the results of the search and selection process, from the number of records identified in the search to the number of studies included in the review, ideally using a flow diagram. | Section 3,  1^st^ paragraph |
|  | 16b | Cite studies that might appear to meet the inclusion criteria, but which were excluded, and explain why they were excluded. | Section 3,  1^st^ paragraph |
| Study characteristics | 17 | Cite each included study and present its characteristics. | Section 3.1  Section 3.3,  1^st^ paragraph and  Appendix E |
| Risk of bias in studies | 18 | Present assessments of risk of bias for each included study. | Section 3.2 and Appendix D |
| Results of individual studies | 19 | For all outcomes, present, for each study: (a) summary statistics for each group (where appropriate) and (b) an effect estimate and its precision (e.g. confidence/credible interval), ideally using structured tables or plots. | Appendix E and  Sections 3.3‒3.5 |
| Results of syntheses | 20a | For each synthesis, briefly summarise the characteristics and risk of bias among contributing studies. | Sections 3.3‒3.5 |
|  | 20b | Present results of all statistical syntheses conducted. If meta-analysis was done, present for each the summary estimate and its precision (e.g. confidence/credible interval) and measures of statistical heterogeneity. If comparing groups, describe the direction of the effect. | NA |
|  | 20c | Present results of all investigations of possible causes of heterogeneity among study results. | NA |
|  | 20d | Present results of all sensitivity analyses conducted to assess the robustness of the synthesized results. | NA |
| Reporting biases | 21 | Present assessments of risk of bias due to missing results (arising from reporting biases) for each synthesis assessed. | NA |
| Certainty of evidence | 22 | Present assessments of certainty (or confidence) in the body of evidence for each outcome assessed. | NA |
| **DISCUSSION** | | |  |
| Discussion | 23a | Provide a general interpretation of the results in the context of other evidence. | Section 4 |
|  | 23b | Discuss any limitations of the evidence included in the review. | Section 4 |
|  | 23c | Discuss any limitations of the review processes used. | Section 4 |
|  | 23d | Discuss implications of the results for practice, policy, and future research. | Section 4 |
| **OTHER INFORMATION** | | |  |
| Registration and protocol | 24a | Provide registration information for the review, including register name and registration number, or state that the review was not registered. | NA |
|  | 24b | Indicate where the review protocol can be accessed, or state that a protocol was not prepared. | NA |
|  | 24c | Describe and explain any amendments to information provided at registration or in the protocol. | NA |
| Support | 25 | Describe sources of financial or non-financial support for the review, and the role of the funders or sponsors in the review. | Section “Funding” |
| Competing interests | 26 | Declare any competing interests of review authors. | Section “Competing interests” |
| Availability of data, code and other materials | 27 | Report which of the following are publicly available and where they can be found: template data collection forms; data extracted from included studies; data used for all analyses; analytic code; any other materials used in the review. | Section  “Supplementary data” |

^†^Page MJ, McKenzie JE, Bossuyt PM, Boutron I, Hoffmann TC, Mulrow CD, et al. The PRISMA 2020 statement: An updated guideline for reporting systematic reviews. *BMJ (Online)*, *372*, n71–n71. <https://doi.org/10.1136/bmj.n71>

## Supplementary Table 2. Excluded studies.

| 1 | Arbesman, M., Bazyk, S., & Nochajski, S. M. Systematic review of occupational therapy and mental health promotion, prevention, and intervention for children and youth. The American Journal of Occupational Therapy (2013) 67:6, e120–e130. <https://doi.org/10.5014/ajot.2013.008359> |
| --- | --- |
| 2 | Awa P, Plaumann M, Walter U. Burnout prevention: A review of intervention programs. Patient Education and Counseling (2010) 78:2, 184–190. <https://doi.org/10.1016/j.pec.2009.04.008>. |
| 3 | Bajaj, M. K., Malhotra, Sunita. Micro and macro level intervention in promotion of mental health: Some evidences. Indian Journal of Community Psychology (2009) 5:2, pp. 235-246. |
| 4 | Carolan, S., Harris, P. R., & Cavanagh, K. Improving Employee Well-Being, and Effectiveness: Systematic Review and Meta-Analysis of Web-Based Psychological Interventions Delivered in the Workplace. Journal of Medical Internet Research (2017) 19:7, e271–e271. <https://doi.org/10.2196/jmir.7583> |
| 5 | Castillo, E. G., Ijadi-Maghsoodi, R., Shadravan, S., Moore, E., Mensah, M. O., Docherty, M., et al. Community Interventions to Promote Mental Health and Social Equity. Current Psychiatry Reports (2019) 21:5, 35–35. <https://doi.org/10.1007/s11920-019-1017-0> |
| 6 | Cavaleri, M. A., Olin, S. S., Kim, A., Hoagwood, K. E., & Burns, B. J. Family Support in Prevention Programs for Children at Risk for Emotional/Behavioral Problems. Clinical Child and Family Psychology Review (2011) 14:4, 399–412. <https://doi.org/10.1007/s10567-011-0100-9> |
| 7 | Codjoe, L., Barber, S., Ahuja, S., Thornicroft, G., Henderson, C., Lempp, H., & N’Danga-Koroma, J. Evidence for interventions to promote mental health and reduce stigma in Black faith communities: systematic review. Social psychiatry and psychiatric epidemiology (2021) 56:6, 895–911. <https://doi.org/10.1007/s00127-021-02068-y> |
| 8 | Conley, C. S., Durlak, J. A., & Dickson, D. A. An Evaluative Review of Outcome Research on Universal Mental Health Promotion and Prevention Programs for Higher Education Students. Journal of American College Health (2013) 61:5, 286–301. <https://doi.org/10.1080/07448481.2013.802237> |
| 9 | Czabała, C., Charzyńska, K., & Mroziak, B. Psychosocial interventions in workplace mental health promotion: an overview. Health Promotion International (2011) 26(suppl_1), i70–i84. <https://doi.org/10.1093/heapro/dar050> |
| 10 | Domingues, R. B. Modern postural yoga as a mental health promoting tool: A systematic review. Complementary Therapies in Clinical Practice (2018) 31, 248–255. [https://doi.org/10.1016/j.ctcp.2018.03.002](https://doi.org/10.1016/j.ctcp.2018.03.002D'Onofrio02) |
| 11 | D’Onofrio, G., & Degutis, L. C. Preventive Care in the Emergency Department: Screening and Brief Intervention for Alcohol Problems in the Emergency Department: A Systematic Review. Academic Emergency Medicine (2002) 9(6), 627–638. <https://doi.org/10.1197/aemj.9.6.627> |
| 12 | Duhoux, A., Menear, M., Charron, M., Lavoie‐Tremblay, M., & Alderson, M. Interventions to promote or improve the mental health of primary care nurses: a systematic review. Journal of Nursing Management (2017) 25(8), 597–607. <https://doi.org/10.1111/jonm.12511> |
| 13 | Enns J, Holmqvist M, Wener P. et al. Mapping interventions that promote mental health in the general population: A scoping review of reviews. Preventive Medicine (2016) 87:70–80. <http://dx.doi.org/10.1016/j.ypmed.2016.02.022> |
| 14 | Fenwick-Smith, A., Dahlberg, E. E., & Thompson, S. C. Systematic review of resilience-enhancing, universal, primary school-based mental health promotion programs. BMC Psychology (2018) 6:1, 30–30. <https://doi.org/10.1186/s40359-018-0242-3> |
| 15 | Fernandez, A., Howse, E., Rubio-Valera, M., Thorncraft, K., Noone, J., Luu, X., et al. Setting-based interventions to promote mental health at the university: a systematic review. International Journal of Public Health (2016) 61:7, 797–807. <https://doi.org/10.1007/s00038-016-0846-4> |
| 16 | Gray, P., Senabe, S., Naicker, N., Kgalamono, S., Yassi, A., & Spiegel, J. M. Workplace-Based Organizational Interventions Promoting Mental Health and Happiness among Healthcare Workers: A Realist Review. International Journal of Environmental Research and Public Health (2019) 16:22, 4396–. <https://doi.org/10.3390/ijerph16224396> |
| 17 | Gould, T., Parker, E., & Clelland, N. Searching for evidence: what works in Indigenous mental health promotion? Health Promotion Journal of Australia (2007) 18:3, 208–216. <https://doi.org/10.1071/HE07208> |
| 18 | Herrman, H. The need for mental health promotion. Australasian Psychiatry : Bulletin of the Royal Australian and New Zealand College of Psychiatrists (2001) 35:6, 709–715. <https://doi.org/10.1046/j.1440-1614.2001.00947.x> |
| 19 | Hirst, S. P., Lane, A., & Stares, R. Health Promotion With Older Adults Experiencing Mental Health Challenges: A Literature Review of Strength-Based Approaches. Clinical Gerontologist (2013) 36:4, 329–355. <https://doi.org/10.1080/07317115.2013.788118> |
| 20 | Hosker, D. K., Elkins, R. M., & Potter, M. P. Promoting Mental Health and Wellness in Youth Through Physical Activity, Nutrition, and Sleep. Child and Adolescent Psychiatric Clinics of North America (2019) 28:2, 171–193. <https://doi.org/10.1016/j.chc.2018.11.010> |
| 21 | Jané-Llopis E., Katschnig, H., McDaid, D., & Wahlbeck, K. Supporting decision-making processes for evidence-based mental health promotion. Health Promotion International (2011) 26(suppl_1), i140–i146. <https://doi.org/10.1093/heapro/dar076> |
| 22 | Kaminski, J. W., Robinson, L. R., Hutchins, H. J., Newsome, K. B., & Barry, C. M. Evidence base review of couple‐ and family‐based psychosocial interventions to promote infant and early childhood mental health, 2010–2019. Journal of Marital and Family Therapy (2022) 48:1, 23–55. <https://doi.org/10.1111/jmft.12570> |
| 23 | Kuosmanen, T., Clarke, A. M., & Barry, M. M. Promoting adolescents’ mental health and wellbeing: evidence synthesis. Journal of Public Mental Health (2019) 18:1, 73–83. <https://doi.org/10.1108/JPMH-07-2018-0036> |
| 24 | Kutcher, S., & Wei, Y. Mental health and the school environment: secondary schools, promotion and pathways to care. Current Opinion in Psychiatry (2012) 25:4, 311–316. <https://doi.org/10.1097/YCO.0b013e3283543976> |
| 25 | Leijten, P., Gardner, F., Melendez-Torres, G., Van Aar, J., Hutchings, J., Schulz, S., et al. Meta-analyses: key parenting program components for disruptive child behavior. Journal of the American Academy of Child and Adolescent Psychiatry (2019) 58:2, 180–190. <https://doi.org/10.1016/j.jaac.2018.07.900> |
| 26 | Lesesne, C. A., & Kennedy, C. Starting Early: Promoting the Mental Health of Women and Girls Throughout the Life Span. Journal of Women’s Health (Larchmont, N.Y. 2002) (2005) 14:9, 754–763. <https://doi.org/10.1089/jwh.2005.14.754> |
| 27 | Li, C., Yin, H., Zhao, J., Shang, B., Hu, M., Zhang, P., & Chen, L. Interventions to promote mental health in nursing students: A systematic review and meta‐analysis of randomized controlled trials. Journal of Advanced Nursing (2018) 74:12, 2727–2741. <https://doi.org/10.1111/jan.13808> |
| 28 | Nair, B., & Otaki, F. Promoting University Students’ Mental Health: A Systematic Literature Review Introducing the 4M-Model of Individual-Level Interventions. Frontiers in Public Health (2021) 9, 699030–699030. <https://doi.org/10.3389/fpubh.2021.699030> |
| 29 | Nilsen, P. Brief alcohol intervention to prevent drinking during pregnancy: an overview of research findings. Current Opinion in Obstetrics & Gynecology (2009) 21:6, 496–500. <https://doi.org/10.1097/GCO.0b013e328332a74c> |
| 30 | O’Connor, C. A., Dyson, J., Cowdell, F., & Watson, R. Do universal school‐based mental health promotion programmes improve the mental health and emotional wellbeing of young people? A literature review. Journal of Clinical Nursing (2018) 27:3-4, e412–e426. <https://doi.org/10.1111/jocn.14078> |
| 31 | O’Reilly, M., Svirydzenka, N., Adams, S., & Dogra, N. Review of mental health promotion interventions in schools. Social Psychiatry and Psychiatric Epidemiology (2018) 53:7, 647–662. <https://doi.org/10.1007/s00127-018-1530-1> |
| 32 | Reavley, N., & Jorm, A. F. Prevention and early intervention to improve mental health in higher education students: a review. Early Intervention in Psychiatry (2010) 4:2, 132–142. <https://doi.org/10.1111/j.1751-7893.2010.00167.x> |
| 33 | Ruiz‐Casares, M., Drummond, J. D., Beeman, I., & Lach, L. M. Parenting for the promotion of adolescent mental health: a scoping review of programmes targeting ethnoculturally diverse families. Health & Social Care in the Community (2017) 25:2, 743–757. <https://doi.org/10.1111/hsc.12364> |
| 34 | Salazar de Pablo, G., De Micheli, A., Nieman, D. H., Correll, C. U., Kessing, L. V., Pfennig, A., et al. Universal and selective interventions to promote good mental health in young people: Systematic review and meta-analysis. European Neuropsychopharmacology (2020) 41, 28–39. <https://doi.org/10.1016/j.euroneuro.2020.10.007> |
| 35 | Seaton, C. L., Bottorff, J. L., Jones-Bricker, M., Oliffe, J. L., DeLeenheer, D., & Medhurst, K. (2017). Men’s Mental Health Promotion Interventions: A Scoping Review. American Journal of Men’s Health (2017) 11:6, 1823–1837. <https://doi.org/10.1177/1557988317728353> |
| 36 | Sharma, A., Sharma, S. D., & Sharma, M. Mental health promotion: a narrative review of emerging trends. Current Opinion in Psychiatry (2017) 30:5, 339–345. <https://doi.org/10.1097/YCO.0000000000000347> |
| 37 | Skara, S., & Sussman, S. A review of 25 long-term adolescent tobacco and other drug use prevention program evaluations. Preventive Medicine (2003) 37:5, 451–474. <https://doi.org/10.1016/S0091-7435(03)00166-X> |
| 38 | Tan L, Wang M-J, Modini M et al. Preventing the development of depression at work: a systematic review and meta-analysis of universal interventions in the workplace. BMC Medicine (2014) 12:74. <http://www.biomedcentral.com/1741-7015/12/74> |
| 39 | Toumbourou, J. W., Hemphill, S. A., Tresidder, J., Humphreys, C., Edwards, J., & Murray, D. Mental health promotion and socio‐economic disadvantage: lessons from substance abuse, violence and crime prevention and child health. Health Promotion Journal of Australia (2007) 18:3, 184–190. <https://doi.org/10.1071/HE07184> |
| 40 | Tsutsumi, A. Development of an Evidence-based Guideline for Supervisor Training in Promoting Mental Health: Literature Review. Journal of occupational health (2011) 53:1, 1–9. <https://doi.org/10.1539/joh.R10002> |
| 41 | Vanhove H, Herian M, Perez A et al. Can resilience be developed at work? A meta-analytic review of resilience-building program effectiveness. Journal of Occupational and Organizational Psychology (2016) 89:2, 278–307. <https://doi.org/10.1111/joop.12123> |
| 42 | Wells, J., Barlow, J., & Stewart-Brown, S. A systematic review of universal approaches to mental health promotion in schools. Health Education (Bradford, West Yorkshire, England) (2003) 103:4, 197–220. <https://doi.org/10.1108/09654280310485546> |
| 43 | Zechmeister, I., Kilian, R., & McDaid, D. Is it worth investing in mental health promotion and prevention of mental illness? A systematic review of the evidence from economic evaluations. BMC Public Health (2008) 8:1, 20–20. <https://doi.org/10.1186/1471-2458-8-20> |

## Supplementary Table 3. The methodological quality of included systematic reviews using AMSTAR 2 criteria^‡^

|  | Q1. PICO | **Q2. A priori design*** | Q3. Study Design | **Q4. Search*** | Q5. Study Selection | Q6. Data Extraction | **Q7. Excluded Studies*** | Q8. Included Studies | **Q9. Risk of Bias (RoB) assessment*** | Q10. Funding (of Included studies) | **Q11. Statistical Methods*** | Q12. Impact of RoB on Results | **Q13. RoB in Interpretation*** | Q14. Heterogeneity | **Q15. Publication Bias*** | Q16. Conflict of Interest | **Overall rating**  **(max. 16);**  **Weaknesses**** |
| --- | --- | --- | --- | --- | --- | --- | --- | --- | --- | --- | --- | --- | --- | --- | --- | --- | --- |
| Barlow, 2014(20) | Yes | **Yes** | Yes | **Yes** | Yes | Yes | **Yes** | Yes | **Yes** | No | **Yes** | Yes | **Yes** | Yes | **No** | Yes | **14; 1** |
| Bartlett, 2019(21) | Yes | **Yes** | Yes | **Yes** | Yes | Yes | **No** | Yes | **Yes** | No | **Yes** | Yes | **Yes** | Yes | **Yes** | Yes | **14; 1** |
| Bhui, 2012(22) | No | **No** | Yes | **Yes** | Yes | Yes | **No** | Yes | **No** | No | **NA** | NA | **No** | No | **NA** | Yes | **6; 4** |
| Bowler, 2010(23) | No | **Yes** | Yes | **Yes** | Yes | Yes | **No** | Yes | **Yes** | No | **Yes** | Yes | **Yes** | Yes | **Yes** | Yes | **13; 1** |
| Clarke, 2015(24) | No | **P Yes** | Yes | **P Yes** | Yes | Yes | **No** | Yes | **Yes** | No | **NA** | NA | **Yes** | No | **NA** | Yes | **9; 1** |
| Conley, 2015(25) | No | **No** | No | **P Yes** | No | No | **No** | Yes | **No** | No | **Yes** | NA | **NA** | No | **No** | Yes | **4; 4** |
| Dawson, 2020(26) | No | **Yes** | Yes | **Yes** | Yes | Yes | **No** | Yes | **Yes** | No | **Yes** | Yes | **Yes** | Yes | **Yes** | No | **12; 1** |
| Galante, 2021(27) | Yes | **Yes** | Yes | **Yes** | Yes | Yes | **No** | Yes | **Yes** | No | **Yes** | Yes | **Yes** | Yes | **Yes** | Yes | **14; 1** |
| Gottlieb, 2011(28) | No | **No** | Yes | **Yes** | Yes | Yes | **No** | Yes | **No** | No | **NA** | NA | **NA** | No | **NA** | Yes | **6; 3** |
| Hunter, 2019(29) | No | **Yes** | Yes | **Yes** | Yes | Yes | **No** | Yes | **Yes** | No | **NA** | NA | **Yes** | No | **NA** | Yes | **9; 1** |
| Koopman, 2017(30) | No | **No** | No | **Yes** | Yes | Yes | **No** | Yes | **Yes** | No | **NA** | Yes | **Yes** | No | **NA** | Yes | **8; 2** |
| Lampert, 2021(31) | No | **No** | No | **Yes** | Yes | Yes | **No** | Yes | **Yes** | No | **NA** | NA | **Yes** | NA | **NA** | Yes | **7; 2** |
| Le, 2021(32) | Yes | **Yes** | Yes | **Yes** | Yes | Yes | **No** | Yes | **Yes** | No | **NA** | NA | **Yes** | No | **NA** | Yes | **10; 1** |
| Lo, 2018(33) | Yes | **No** | No | **Yes** | Yes | Yes | **No** | Yes | **Yes** | No | **Yes** | Yes | **Yes** | Yes | **No** | No | **10; 3** |
| Macedo, 2014(34) | No | **No** | No | **Yes** | No | No | **No** | Yes | **Yes** | No | **NA** | NA | **No** | No | **NA** | Yes | **4; 3** |
| Mammen & Faulkner, 2013(35) | No | **No** | No | **Yes** | No | No | **No** | Yes | **Yes** | No | **NA** | NA | **Yes** | No | **NA** | Yes | **5; 2** |
| Otto, 2021(36) | Yes | **Yes** | No | **Yes** | Yes | Yes | **Yes** | Yes | **Yes** | No | **NA** | NA | **No** | Yes | **NA** | Yes | **10; 1** |
| Pieper, 2019(37) | No | **No** | No | **Yes** | Yes | Yes | **No** | No | **Yes** | No | **NA** | NA | **Yes** | Yes | **NA** | Yes | **7; 2** |
| Proper, 2019(38) | No | **No** | No | **Yes** | Yes | Yes | **No** | Yes | **Yes** | No | **NA** | NA | **Yes** | Yes | **NA** | Yes | **8; 2** |
| Sandler, 2014(39) | No | **No** | Yes | **Yes** | No | No | **No** | Yes | **No** | No | **NA** | NA | **NA** | Yes | **NA** | Yes | **5; 3** |

* = Critical domain; ** = Number of critical domain **weaknesses (max. 7);** NA = Not applicable; P Yes = Partly Yes
^†^Shea BJ, Reeves BC, Wells G, Thuku M, Hamel C, Moran J, et al. AMSTAR 2: A critical appraisal tool for systematic reviews that include randomised or non-randomised studies of healthcare interventions, or both. BMJ. 2017;358.

Q1. Did the research questions and inclusion criteria for the review include the components of PICO?
**Q2. Did the report of the review contain an explicit statement that the review methods were established prior to the conduct of the review and did the report justify any significant deviations from the protocol?***Q3. Did the review authors explain their selection of the study designs for inclusion in the review?
**Q4. Did the review authors use a comprehensive literature search strategy?***Q5. Did the review authors perform study selection in duplicate?
Q6. Did the review authors perform data extraction in duplicate?
**Q7. Did the review authors provide a list of excluded studies and justify the exclusions?***Q8. Did the review authors describe the included studies in adequate detail?
**Q9. Did the review authors use a satisfactory technique for assessing the risk of bias (RoB) in individual studies that were included in the review?***Q10. Did the review authors report on the sources of funding for the studies included in the review?
**Q11. If meta-analysis was performed did the review authors use appropriate methods for statistical combination of results?***Q12. If meta-analysis was performed, did the review authors assess the potential impact of RoB in individual studies on the results of the meta-analysis or other evidence synthesis?
**Q13. Did the review authors account for RoB in individual studies when interpreting/ discussing the results of the review?**Q14. Did the review authors provide a satisfactory explanation for, and discussion of, any heterogeneity observed in the results of the review?
**Q15. If they performed quantitative synthesis did the review authors carry out an adequate investigation of publication bias (small study bias) and discuss its likely impact on the results of the review?***Q16. Did the review authors report any potential sources of conflict of interest, including any funding they received for conducting the review?

**Rating overall confidence in the results of the review**

**• High**No or one non-critical weakness: the systematic review provides an accurate and comprehensive summary of the results of the available studies that address the question of interest.

**• Moderate**More than one non-critical weakness*: the systematic review has more than one weakness but no critical flaws. It may provide an accurate summary of the results of the available studies that were included in the review.

**• Low**One critical flaw with or without non-critical weaknesses: the review has a critical flaw and may not provide an accurate and comprehensive summary of the available studies that address the question of interest.

**• Critically low**More than one critical flaw with or without non-critical weaknesses: the review has more than one critical flaw and should not be relied on to provide an accurate and comprehensive summary of the available studies.

*Multiple non-critical weaknesses may diminish confidence in the review, and it may be appropriate to move the overall appraisal down from moderate to low confidence.

**References of the Supplementary Table 3.**

20. Barlow J, Smailagic N, Huband N, Roloff V, Bennett C. Group-based parent training programmes for improving parental psychosocial health. Cochrane Database of Syst Rev (2014) Issue 5. Art. No.: CD002020. DOI: 10.1002/14651858.CD002020.pub4.

21. Bartlett L, Martin A, Neil AL, Memish K, Otahal P, Kilpatrick M, et al. A systematic review and meta-analysis of workplace mindfulness training randomized controlled trials. J Occup Health Psychol (2019) 24:1. doi: 10.1037/ocp0000146

22. Bhui KS, Dinos S, Stansfeld SA, White PD. A synthesis of the evidence for managing stress at work: A review of the reviews reporting on anxiety, depression, and absenteeism. J Environ Public Health (2012) 2012:515874-21. doi: 10.1155/2012/515874

23. Bowler DE, Buyung-Ali LM, Knight TM, Pullin AS. A systematic review of evidence for the added benefits to health of exposure to natural environments. BMC Public Health (2010) 10:1. doi: 10.1186/1471-2458-10-456

24. Clarke AM, Kuosmanen T, Barry MM. A Systematic Review of Online Youth Mental Health Promotion and Prevention Interventions. J Youth Adolesc (2015) 44:1, 90–113.doi: 10.1007/s10964-014-0165-0

25. Conley CS, Durlak JA, Kirsch AC. A Meta-analysis of Universal Mental Health Prevention Programs for Higher Education Students. Prev Sci (2015) 16:4, 487–507. doi:10.1007/s11121-015-0543-1

26. Dawson AF, Brown WW, Anderson J, Datta B, Donald JN, Hong K, et al. Mindfulness-Based Interventions for University Students: A Systematic Review and Meta-Analysis of Randomised Controlled Trials. Appl Psychol Health Well Being (2020) 12:2. doi: 10.1111/aphw.12188

27. Galante J, Friedrich C, Dawson AF, Modrego-Alarcón M, Gebbing P, Delgado-Suárez I, et al. Mindfulness-based programmes for mental health promotion in adults in nonclinical settings: A systematic review and meta-analysis of randomised controlled trials. PLoS Med (2021) 18:1. doi: 10.1371/journal.pmed.1003481

28. Gottlieb L, Waitzkin H, Miranda J. Depressive symptoms and their social contexts: A qualitative systematic literature review of contextual interventions. Int J Soc Psychiatry (2011) 57(4): 402–417. doi: 10.1177/0020764010362863

29. Hunter RF, Cleland C, Cleary A, Droomers M, Wheeler BW, Sinnett D, et al. Environmental, health, wellbeing, social and equity effects of urban green space interventions: A meta-narrative evidence synthesis. Environ Int (2019) 130, p.104923. doi: 10.1016/j.envint.2019.104923

30. Koopman MY, Pieterse ME, Bohlmeijer ET, Drossaert CHC. Mental health promoting Interventions for the unemployed: a systematic review of applied techniques and effectiveness. Int J Ment Health Promot (2017) 19:4. doi: 10.1080/14623730.2017.1328367

31. Lampert T, Costa J, Santos O, Sousa J, Ribeiro T, Freire E. Evidence on the contribution of community gardens to promote physical and mental health and well-being of non-institutionalized individuals: A systematic review. PLoS ONE (2021) 16:8. doi: 10.1371/journal.pone.0255621

32. Le LKD, Esturas AC, Mihalopoulos C, Chiotelis O, Bucholc J, Chatterton M Lou, et al. Cost-effectiveness evidence of mental health prevention and promotion interventions: A systematic review of economic evaluations. PLoS Med (2021) 18:5. doi: 10.1371/journal.pmed.1003606

33. Lo K, Waterland J, Todd P, Gupta T, Bearman M, Hassed C, et al. Group interventions to promote mental health in health professional education: a systematic review and meta-analysis of randomised controlled trials. Adv Health Sci Educ Theory Pract (2018) 23:2. doi: 10.1007/s10459-017-9770-5

34. Macedo T, Wilheim L, Gonçalves R, Coutinho ESF, Vilete L, Figueira I, et al. Building resilience for future adversity: A systematic review of interventions in non-clinical samples of adults. BMC Psychiatry (2014) 14:1. doi: 10.1186/s12888-014-0227-6

35. Mammen G, Faulkner G. Physical activity and the prevention of depression: A systematic review of prospective studies. Am J Prev Med (2013) 45:5. doi: 10.1016/j.amepre.2013.08.001

36. Otto AK, Gutsch C, Bischoff LL, Wollesen B. Interventions to promote physical and mental health of nurses in elderly care: A systematic review. Prev Med (2021) 148, p.106591. doi: 10.1016/j.ypmed.2021.106591

37. Pieper C, Schröer S, Eilerts AL. Evidence of workplace interventions-A systematic review of systematic reviews. Int J Environ Res Public Health (2019) 16:19. doi: 10.3390/ijerph16193553

38. Proper KI, Van Oostrom SH. The effectiveness of workplace health promotion interventions on physical and mental health outcomes – A systematic review of reviews. Scand J Work Environ Health (2019) 45:6. 10.5271/sjweh.3833

39. Sandler I, Wolchi SA, Cruden G, Mahrer NE, Ahn S, Brincks A, et al. Overview of meta-analyses of the prevention of mental health, substance use, and conduct problems. Annu Rev Clin Psychol (2014) 10:1. doi: 10.1146/annurev-clinpsy-050212-185524
